# Trafficking of central opioid receptors and descending pain inhibition

**DOI:** 10.1186/1744-8069-3-37

**Published:** 2007-12-04

**Authors:** Bihua Bie, Zhizhong Z Pan

**Affiliations:** 1 Department of Anesthesiology and Pain Medicine, The University of Texas-MD Anderson Cancer Center, 1515 Holcombe Boulevard, Houston, TX 77030, USA

## Abstract

The delta-opioid receptor (DOR) belongs to the superfamily of G-protein-coupled receptors (GPCRs) with seven transmembrane domains, and its membrane trafficking is regulated by intracellular sorting processes involving its C-tail motifs, intracellular sorting proteins, and several intracellular signaling pathways. In the quiescent state, DOR is generally located in the intracellular compartments in central neurons. However, chronic stimulation, such as chronic pain and sustained opioid exposure, may induce membrane trafficking of DOR and its translocation to surface membrane. The emerged functional DOR on cell membrane is actively involved in pain modulation and opioid analgesia. This article reviews current understanding of the mechanisms underlying GPCRs and DOR membrane trafficking, and the analgesic function of emerged DOR through membrane trafficking under certain pathophysiological circumstances.

## Introduction

Currently, opioids are still the most effective analgesics available in the clinical treatment of moderate and severe pain. Pharmacological and molecular cloning studies have clearly established three classic types of opioid receptors, μ (MOR), δ, and κ (KOR), which belong to the superfamily of GPCRs with seven transmembrane domains. All three opioid receptors are involved in major opioid actions, including analgesia, reward, and the development of analgesic tolerance and physical dependence [1]. At present, most clinically used opioids for analgesia are either nonselective opioid agonists or selective MOR agonists. The analgesic efficacy of currently used opioid analgesics is generally limited due to their tendency to cause analgesic tolerance, physical dependence and other undesirable side effects after prolonged and repeated use.

Traditionally, DOR agonists have been regarded as very weak analgesics with minimal potential of being used clinically for pain relief although they may produce fewer undesirable side effects than MOR agonists [2]. In animal studies, the analgesic effect of DOR agonists has not been consistently clarified. In opioid naïve animals, although activation of DOR in the spinal cord produces antinociception [3], the effect of DOR agonists applied in the supraspinal sites is inconsistent. Microinjection of DOR agonists into the nucleus raphe magnus (NRM) in the medulla or periaqueductal gray (PAG) in the midbrain produces little or weak antinociceptive effects in normal animals [4-7]. However, a significant antinociceptive effect mediated by DOR in the NRM through its descending pain-modulating system has been reported recently in rats after repeated morphine treatment [4]. The lack of DOR analgesia in normal conditions has been largely attributed to the normally intracellular localization of DOR. Interestingly, several behavioral conditions have been found to induce DOR membrane trafficking and enhance DOR analgesia, including chronic inflammatory or neuropathic pain [8-12], prolonged morphine treatment [3,4,11,13-15], and stress [16].

As a member of the family A GPCRs, DOR, once synthesized intracellularly or internalized upon agonist stimulation, is subject to the process of intracellular sorting through either proteolysis in lysosomes or recycling to cell surface. Unlike MOR, which is normally expressed on the cell surface membrane, DOR is predominantly located in the intracellular compartments in most central neurons under normal conditions [17-19]. However, recent evidence has shown that certain stimuli, such as DOR agonists and chronic pain, can trigger intracellular signals to promote the sorting pathway for DOR membrane trafficking, and enable the emerged DOR on surface membrane to exert physiological functions. Here in this review, we summarize recent reports of DOR membrane trafficking and its functional significance, including basic processes and modulation of DOR membrane trafficking, and behavioral conditions that may induce the membrane trafficking of DOR, and particularly, the DOR in the brainstem for descending inhibition of pain.

## Mechanisms of opioid receptor trafficking

### Basic concepts of GPCR trafficking

**Figure 1:**
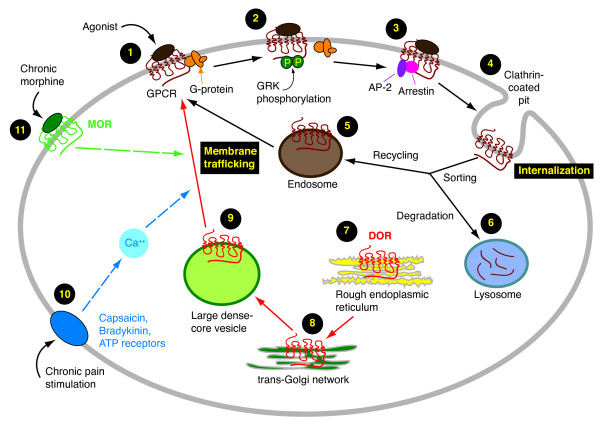
Mechanisms of membrane trafficking for G-protein-coupled receptors (GPCRs). Upon agonist binding (**1**), a GPCR, including the delta-opioid receptor (DOR), is phosphorylated by GPCR kinases (GRK) (**2**), binds to proteins AP-2 and arrestin (**3**), and undergoes the process of internalization via endocytosis through clathrin-coated pit (**4**). Once internalized, the receptor is subjected to highly regulated sorting processes and is targeted either to endosomes in the recycling pathway (**5**) for membrane insertion, or to lysosomes for degradation through the degradation pathway (**6**). DOR is synthesized in the endoplasmic reticulum (ER) (**7**), then transported to the trans-Golgi network (**8**) through ER-Golgi complex, and becomes a mature receptor. Matured DOR is normally targeted intracellularly in large dense-core vesicles (**9**) as intracellular pool of DOR ready for membrane trafficking and insertion. Chronic pain conditions induce the release of a number of inflammatory mediators, which activate corresponding receptors (**10**) and increase intracellular calcium concentration, causing the membrane trafficking of DOR. Persistent stimulation of mu-opioid receptors (MOR) by chronic opioids (**11**) can induce the membrane trafficking of intracellular DOR and bring out functional DOR through yet unknown mechanisms.

Upon agonist binding and stimulation, most GPCRs undergo phosphorylation and internalization through clathrin-coated pits [19] (Figure 1). Once internalized, the receptor may undertake either of the two trafficking fates: being rapidly recycled back to the plasma membrane (recycling pathway), or being targeted to lysosomes for proteolysis (degradative pathway) [20,21]. In the recycling pathway, which is the default trafficking route for MOR, β_2_-adrenergic receptors and NK1 receptors, the receptor dissociates from the ligand in the acidic pH of the endosomal compartment, is dephosphorylated and subsequently returned to the plasma membrane. By contrast, in the lysosomal pathway, which is the preferred sorting pathway for DOR and the protease-activated receptor 1 (PAR-1), the receptor is targeted for degradation in lysosomes. The process of intracellular sorting of GPCRs to recycling endosomes or lysosomal degradation compartments involves complex protein-protein interactions, and is subjected to modulations through intrinsic receptor motifs, intracellular signaling pathways and several protein kinases. These regulatory factors essentially control the rate of receptor internalization, recycling or lysosomal degradation, and consequently the magnitude and duration of receptor signaling.

### Structural determinants of GPCR trafficking related to intracellular degradation

Accumulating evidence has revealed that the final fates of intracellular GPCRs, either to surface membrane for recycling or to lysosomes for degradation, are crucially determined by intrinsic recognition mechanisms between specific motifs in the cytoplasmic domains of GPCRs, particularly in the carboxyl terminus, and intracellular interacting adaptors named sorting proteins [22,23]. For DOR, it is believed that the interaction between the cytoplasmic carboxyl tail and certain sorting proteins, such as GPCR-associated sorting protein (GASP) [23,24], may lead the receptor sorting to the default degradation pathway under naïve conditions.

Similar to other GPCRs, DOR has a common molecular topology including a hydrophobic core of seven membrane-spanning α-helices, three intracellular loops, three extracellular loops, an extracellular amino terminus, and an intracellular carboxyl terminus [1]. A small conserved region within the cytoplasmic carboxyl tail serves as a critical recognition site for DOR interaction with sorting proteins, such as GASP, in the determination of its lysosomal sorting and proteolysis after agonist stimulation and internalization [22-24]. This carboxyl terminal containing the conserved motif NPXXY has higher binding affinity for the lysosome-targeting sorting proteins GASP and sorting nexin-1 (SNX-1), but relatively lower binding affinity for the recycling sorting protein N-ethylmaleimide-sensitive factor (NSF), and even undetectable binding affinity for the recycling sorting protein Ezrin-radixin-moesin (ERM)-binding phosphoprotein-50/Na^+^/H^+ ^exchanger regulatory factor-1 (EBP50/NHERF-1) [22,24]. Conversely, the carboxyl terminal of β_2_-adrenergic receptors shows higher affinity for the recycling sorting proteins EBP50/NHERF and NSF, but lower affinity for GASP and SNX-1 [22]. The interaction between the carboxyl terminal of GPCRs and specific sorting proteins may critically determine the sorting fate of intracellular GPCRs. For example, chimeric exchange of the C-terminus of MOR and DOR has been shown to dramatically divert the sorting fate of these two opioid receptors [23].

### Sorting proteins

Sorting proteins of GPCRs are a number of intracellular adaptors or scaffolding proteins proposed to govern the differential sorting events, and generally have relatively high affinity for the carboxyl terminus of GPCRs. Sorting proteins decisively influence the post-endocytic fate of GPCRs via interaction with the C-tail domains of a receptor, which is well exemplified by the sorting protein GASP that determines the lysosomal sorting of DOR [23] and dopamine D_2 _receptors [25]. GASP is an intracellular protein with 1395 residues and belongs to a novel family of proteins containing a conserved 250-residue carboxyl terminal domain [24]. Disrupting the interaction between GASP and the carboxyl terminal tail of DOR or D_2 _receptors reroutes their post-endocytic sorting from normally a degradative lysosomal fate to the recycling pathway [23,25]. Meanwhile, chimeric studies have revealed that replacement of the sequence in the C-tail of DOR with the corresponding sequence of MOR is sufficient to impart the recycling property of MOR for DOR in HEK293 cells [23]. Taken together, these findings identify GASP as a key protein to determine the degradative fate of some GPCRs, including DOR.

Similarly, SNX-1 is another candidate sorting protein involved in targeting GPCRs, including DOR, to the degradative pathway [20,22], as it has a low affinity for GPCRs that prefer the recycling pathway [26]. It serves as a key determinant for PAR-1 that is preferentially targeted to lysosomes. Other candidates of lysosome targeting sorting proteins include ubiquitination [20] and rab7 [20,27,28].

In contrast, other sorting proteins, such as EBP50/NHERF-1, promote intracellular GPCRs to the recycling pathway. EBP50/NHERF-1 contains two PSD-95/Disc-large/ZO-1 homology (PDZ) domains and one ERM domain. PDZ domains bind to the C-terminal of GPCRs and ERM domain interacts with intracellular actin cytoskeleton [27], thereby mediating the trafficking of targeted proteins to plasma membrane. Previous studies have established that binding of the PDZ domains of EBP50/NHERF-1 with the C-terminal of certain GPCRs, including KOR and β_2_-adrenergic receptors [27,29], may direct their membrane trafficking. Similarly, NSF [30] and Rab GTPases [28] also serve as the sorting proteins to mediate membrane trafficking of intracellular GPCRs. However, it is currently unknown what recycling sorting proteins are involved in the diversion of DOR trafficking from a normal lysosomal fate to the recycling pathway under certain behavioral conditions mentioned above.

### Endoplasmic reticulum (ER) – Golgi apparatus network-disturbing agents

Intracellular GPCRs, particularly DOR that is normally targeted intracellularly, are synthesized, folded in the ER and then packaged into ER-derived vesicles. These transport vesicles carrying cargo receptors then migrate from the ER to the ER-Golgi intermediate complex, the Golgi apparatus and the trans-Golgi network (TGN) (Figure 1). During this process, receptors undergo post-translational modifications (e.g. glycosylation) to attain mature status. Thereafter, the mature receptors, under precise regulation by intracellular signals, move from the TGN to the plasma membrane via large dense-core vesicles (LDCVs) through yet unidentified mechanisms. Any manipulations disrupting the functions of ER-TGN network may significantly influence the membrane trafficking of GPCRs. Brefeldin A, a Golgi-disturbing agent [31], is capable of diminishing MOR membrane insertion induced by activation of the cAMP-PKA pathway in brainstem neurons [32] as well as DOR membrane trafficking induced by neurotrophin in PC12 cells [18].

### Bradykinin and inflammatory mediators

Bradykinin is a proinflammatory mediator involved in a series of pathophysiological processes including chronic pain. It exerts most of its biological effects by interacting with two classes of GPCRs termed as B1 and B2. The intracellular signaling pathway activated by bradykinin via B2 receptors exhibits the potency to induce membrane insertion of intercellular DOR as measured by DOR inhibition of both the evoked neuropeptide exocytosis and the stimulated adenylyl cyclase activity in cultured rat trigeminal ganglion neurons; these actions of bradykinin are mediated through the protein kinase C (PKC)-dependent signaling pathway [17]. Furthermore, other proinflammatory agents, such as ATP and capsaicin, are also shown to induce DOR insertion into the plasma membrane in dorsal root ganglion (DRG) neurons [33] (Figure 1).

### Substance P

Substance P is the first pronociceptive neuropeptide identified in primary sensory neurons, and is also involved in the modulation of DOR membrane trafficking. The substance P domain of preprotachykinin A (*PPT-A*) directly interacts with the third luminal domain of DOR, and this interaction is required for DOR sorting into LDCVs for membrane insertion in DRG neurons and in cultured cell lines [34,35]. Knockout of *PPT-A *eliminates agonist-induced DOR membrane insertion in isolated DRG neurons, and abolishes analgesia induced by intrathecal injection of a DOR agonist in intact animal [34], confirming the critical involvement of substance P in the process of DOR membrane trafficking in sensory neurons.

### Neurotrophins

Activation of the TrkA signaling pathway by nerve growth factor (NGF) regulates the later (post-ER) events in the anterograde trafficking of intracellular DOR, which is required to maintain the intracellular pool of DOR available for plasma membrane insertion [18]. It has been shown that the cytoplasmic domain in the C-terminal of DOR (27 amino acid residues) contains a signal that determines the specificity of NGF-regulated intracellular targeting of the receptor. Epidermal growth factor (EGF) also can promote the rapid translocation of transient receptor potential (TRP) channel C5, although not a GPCR, inducing its insertion into the plasma membrane via activation of the phosphatidylinositide 3-kinase (PI_3_K) and Rac1, a Rho family GTPase commonly involved in cytoskeletal re-arrangements and initiation of cell morphological changes [36].

### Calcium entry and intracellular calcium store

Because intracellular calcium plays a key role in the trafficking and membrane fusion of protein-containing vesicles, it is conceivable that intracellular calcium signaling is also crucially involved in DOR membrane insertion, especially in the premise that intracellular DOR is predominately located in the LDCVs [33]. In cultured DRG neurons, either depression of extracellular calcium entry or depletion of intracellular inositol (1,4,5)-trisphosphate (IP_3_)-sensitive calcium stores abolishes DOR membrane insertion induced by DOR agonists; meanwhile, the upsurge of intracellular calcium produced by capsaicin, ATP, and high potassium-induced depolarization promotes the membrane insertion of intracellular DOR [18,33,35]. Although the definite role of intracellular calcium in the membrane trafficking of GPCRs has not yet been clearly defined, it is generally postulated that calcium is critically involved, at least, in the process of vesicle transport, cytoskeleton arrangement and membrane fusion.

### Protein kinases

Protein kinases are also found to modulate the trafficking and membrane insertion of GPCRs including DOR. Activation of the cAMP-PKA pathway by the adenylyl cyclase activator forskolin or the nonhydrolysable cAMP analog 8-bromo-cAMP promotes MOR membrane trafficking and consequently MOR inhibition of presynaptic GABA release in rat dorsal motor nucleus of the vagus neurons, an effect blocked by the cAMP-PKA pathway inhibitor H89 [32]. However, forskolin activation of the cAMP-PKA pathway fails to induce functional membrane trafficking of DOR in midbrain PAG neurons [13]. PKC, conceivably involved in membrane trafficking and insertion of glutamate receptors, has also been identified to mediate the bradykinin-induced membrane insertion of DOR in rat sensory neurons [17]. To date, the involvement of other protein kinases, such as MAP kinases and calmodulin kinases, in membrane trafficking of opioid receptors has yet to be explored.

Although many regulatory factors have been identified to critically modulate the intracellular sorting and membrane insertion of GPCRs including DOR in naïve conditions, it remains unknown whether and how these regulatory factors trigger and mediate DOR membrane trafficking induced under several pathophysiological circumstances such as chronic pain and prolonged opioid exposure, as described below.

## Opioid receptor trafficking and pain modulation

### Pain-induced opioid receptor trafficking and pain inhibition

Peripheral sensory neurons in the DRG are nociceptors that receive nociceptive stimuli and deliver the nociceptive information to the modulatory circuits in the spinal dorsal horn. As mentioned above, inflammatory mediators, such as bradykinin [17], substance P [34] and ATP [33], can induce DOR membrane trafficking in cultured sensory neurons *in vitro*. Sustained inflammation induced by complete Freud's adjuvant (CFA) also significantly increases DOR membrane trafficking in small- and medium-sized DRG neurons in intact animals [11]. Local administration of capsaicin, an activator of vanilloid/transient receptor potential vanilloid 1 (TRPV1) selectively located in C-fibers, induces an increase in DOR membrane trafficking in small-sized DRG neurons [11], suggesting that the enhanced membrane recruitment of DOR is tightly adapted to the modality of pain, and may account for the enhanced antinociceptive efficacy of DOR agonists under that condition. Additionally, there is a bilateral upregulation in DOR expression in the DRG neurons of small and large diameters from rats after chronic constriction of the sciatic nerve, resulting in DOR-mediated inhibition of tactile allodynia following nerve injury [8].

The dorsal horn, especially lamina II, of the spinal cord is a critical site for the relay and processing of dynamic sensory information. While spinal administration of DOR agonists induces antinociception in naïve animals [15], DOR-mediated analgesic effects that reverse hyperalgesia and tactile allodynia are dramatically augmented in animals with chronic inflammatory or neuropathic pain [15,37]. Likely, this results from the increased membrane recruitment of DOR in the dorsal horn neurons following the chronic pain. Actually, sustained inflammation induced by CFA is also reported to significantly increase the expression and membrane targeting of DOR in the spinal dorsal horn where the analgesic effect of DOR agonists is largely enhanced [9,10]. This adaptation of DOR during chronic inflammation may require the integrity of MOR as this adaptation is diminished in MOR knockout mice [9]. Also, increased membrane trafficking of functional DOR has been reported in laminas III-VI neurons from rhizotomized rats [14].

Several brain regions including the PAG and NRM are critical sites for supraspinal pain modulation. Pharmacological and electrophysiological evidence has established that the brainstem NRM, receiving major inputs from the PAG, functions as an integral relay in descending modulation of nociception. In these brain regions, DOR is located predominantly in presynaptic axon terminals, rather than on plasma membrane of presynaptic boutons, and immunolabeling for DOR is often associated with intracellular LDCVs [38-40]. In general, the analgesic effect of DOR agonists applied in these two regions is weak in normal animals. Although local microfusion of DOR agonists into the NRM region shows an inhibition of the tail flick-related increase in ON-cell activity and shortens the tail flick-related pause in OFF-cell activity in intact animals [41], microinjection of DOR agonists into either the PAG or NRM has only little or a weak effect on the thermal nociception in normal rats [4,6]. However, persistent inflammation induced by CFA markedly increases the anti-hyperalgesic potency of DOR agonists applied in the NRM, as indicated by a prolonged effect duration and a leftward shift of the dose-response curve with a reduced ED_50 _value, an effect appearing two weeks after inflammatory injury [12]. Also, microinjection of the DOR antagonist naltriben into the NRM enhances the hyperalgesia in the ipsilateral hindpaw, which is at least partially mediated by the increased release of endogenous opioid peptides with preferential affinity for DOR [42]. In addition, microinjection of DOR agonists into the ventral PAG significantly inhibits mechanical allodynia in rats with neuropathic pain [43]. Nevertheless, there is no data currently available regarding the mechanisms for the adaptation and membrane trafficking of DOR induced by chronic pain in the supraspinal sites critically involved in pain modulation.

### Opioid-induced DOR trafficking and opioid analgesia

Peripheral sensory neurons in the DRG are among the critical targets of opioid analgesics acting on opioid receptors, including MOR and DOR, abundantly expressed in the cell body and terminals of DRG neurons [33]. It has been described recently that DOR agonists can rapidly induce the membrane trafficking of intracellular DOR via Ca^2+^-dependent signaling pathways in cultured sensory neurons [33]. Prolonged exposure to morphine (48 hours) also significantly increases DOR membrane trafficking in cultured DRG neurons [11] and cortical neurons [3]. Similarly, sustained systemic treatment with morphine significantly induces the membrane translocation of intracellular DOR in sensory neurons in intact mice [11]. It is believed that the DOR membrane recruitment accounts, at least in part, for the enhanced antinociceptive efficacy of DOR agonists following sustained morphine treatment, and may provide a more effective action site for peripheral analgesics [3].

Spinal dorsal horn, as the primary processing center for nociceptive information, also contains abundant opioid receptors, therefore serving as another critical site for opioid analgesia. DOR in the spinal neurons is mostly, although not exclusively, associated with the intracellular compartments in control conditions [3,44]. Repeated treatment with morphine or other selective MOR agonists induces MOR-dependent membrane insertion of DOR, and increases the bioavailability of DOR in the cultured [3] and *in vivo *[3,14,44] spinal neurons. The increase in functional DOR on surface membrane is thought responsible for the enhanced, DOR agonist-mediated antinociception after chronic opioid treatment [3,44,45].

Through their descending pathways for pain modulation, the brainstem NRM and the midbrain PAG serve as the critical supraspinal sites for opioid analgesia. Despite the abundant expression of DOR in these areas [19,38,39,46], little DOR-mediated cellular actions have been observed under normal conditions, likely due to the intracellular location of these receptors in these two brain regions in naïve animals [4,47]. However, others have reported a DOR-induced weak potassium current in a small population of NRM and PAG neurons [48,49], but DOR agonists have no significant effect on the presynaptic GABA release in these NRM or PAG neurons from normal animals [4,13,49]. Intriguingly, several recent studies have revealed that the intracellular DOR can translocate to the surface membrane and become functional in these neurons from rats chronically treated with morphine [4,13] (Figure 1). In these neurons, DOR agonists elicit a significant inhibition of presynaptic GABA release through activation of the newly inserted, functional DOR, which is absent in normal animals [4,13]. The behavioral significance of this membrane trafficking of DOR in the NRM has been functionally demonstrated by the observations that microinjection of DOR agonists into the NRM, ineffective in opioid naïve animals, produces significant antinociception in chronic morphine-treated animals, and relieves analgesic tolerance to chronic morphine [4]. These observations further support the notion that DOR agonists may be more effective and therefore could serve as better alternative analgesics for pain control following chronic exposure to MOR agonists.

## Conclusion

It is now well documented that DOR is predominantly located in the intracellular compartments in most neurons within the pain-related central circuits in control conditions, and a number of behavioral stimuli, especially chronic pain and prolonged opioid exposure, can induce the membrane trafficking of DOR. The mechanisms underlying membrane trafficking of GPCRs including DOR are still perplexing and poorly understood at present. They may involve precise interactions among the receptor motifs, sorting proteins, inflammatory mediators and intracellular signaling pathways under certain physiological and pathophysiological conditions. Better understanding of the mechanisms and underlying signals and conditions for induction of DOR membrane trafficking would promise the development of more efficacious opioid analgesics with fewer side-effects.

## Competing interests

The author(s) declare that they have no competing interests.

## Authors' contributions

BB drafted the manuscript and ZZP revised the manuscript and drew the figure. Both authors read and approved the final manuscript.
